# Dose and parameter specific effects of curcumin on neuropathic pain, cavity formation, and non-coding RNA expression in a spinal cord injury model

**DOI:** 10.1038/s41598-026-51599-4

**Published:** 2026-05-13

**Authors:** Mohammad Mojtaba Farazi, Maryam Hadadi, Saeideh Jafarinejad-Farsangi, Hamid Najafipour, Marzieh lotfian sargazi, Yousof Mir, Zahra Behroozi

**Affiliations:** 1 Cardiovascular Research Center, Institute of Basic and Clinical Physiology Sciences, Kerman University of Medical Sciences, Kerman, Iran; 2 Physiology Research Center, Institute of Neuropharmacology, Kerman University of Medical Sciences, Kerman, Iran; 3 Endocrinology and Metabolism Research Center, Institute of Basic and Clinical Physiology Sciences, Kerman University of Medical Sciences, Kerman, Iran; 4 Bio Environmental Health Hazards Research Center, Jiroft University of Medical Sciences, Jiroft, Iran

**Keywords:** Spinal cord injury, Curcumin, Neuropathic pain, Non-coding RNAs, MicroRNA, LncRNA, Diseases, Drug discovery, Medical research, Neurology, Neuroscience

## Abstract

Neuropathic pain (NP) represents one of the most frequent complications following spinal cord injury (SCI) and is associated with dysregulation of non-coding RNAs (ncRNAs). Curcumin possesses anti-inflammatory and neuroprotective properties. This study investigated the dose-dependent effects of curcumin—a compound with anti-inflammatory and neuroprotective properties—on pain-related behaviors and ncRNA expression after SCI. Male Wistar rats (*n* = 8 per group) were randomly assigned to 5 groups: Control (no surgery, no treatment), Sham (only laminectomy), SCI (clip-compression injury at the T11–T12 vertebral level), Cur100 and Cur200 (Curcumin at 100 and 200 mg/kg given orally 30 min after SCI for 10 consecutive days). Behavioral tests (acetone drop for cold allodynia, tail-flick for thermal hyperalgesia) were conducted over six weeks. Histological (H&E staining for cavity size) and molecular (qPCR for lncRNAs H19, GAS5, CRNDE and miRNAs miR-21-5p, miR-29a-3p) analyses were performed. Data were analyzed with PRISM software. At the endpoint, SCI induced significant cold allodynia, thermal hyperalgesia along with significant upregulation of all target ncRNAs. Curcumin treatment at both doses attenuated NP and modulated ncRNA expression in a parameter-dependent manner—where effects varied based on the specific outcome measured. The higher dose (200 mg/kg) provided greater neuroprotection and more effectively reduced thermal hyperalgesia, while the lower dose (100 mg/kg) was more potent in alleviating cold allodynia and selectively downregulating pro-inflammatory ncRNAs (H19, GAS5, miR-21-5p). These findings highlight the parameter-specificity of curcumin’s therapeutic effects, suggesting that optimal dosing should be tailored according to the targeted pain modality and molecular pathway.

**Supplementary Information:**

The online version contains supplementary material available at 10.1038/s41598-026-51599-4.

## Introduction

Spinal cord injury (SCI) frequently results in neuropathic pain (NP), a severe and widespread condition affecting up to 70% of patients^1^. NP markedly diminishes patients’ quality of life and is often accompanied by allodynia, hyperalgesia, spontaneous pain, and psychological comorbidities, including depression and anxiety^2,3^. Despite advances in pain management, currently available pharmacological and non-pharmacological interventions remain largely ineffective. As a result, NP imposes a substantial economic burden on healthcare systems, underscoring the urgent need for more effective therapeutic strategies^3^.

In recent years, non-coding RNAs (ncRNAs), including microRNAs (miRNAs) and long non-coding RNAs (lncRNAs), have emerged as key regulators of gene expression and are increasingly recognized for their roles in the pathophysiology of neuropathic pain and other neurological disorders^4–6^. ncRNAs participate in transcriptional and post-transcriptional regulation by interacting with target mRNAs, thereby influencing neuroinflammatory responses, glial activation, and pain-related signaling pathways^6^.

Long non-coding RNAs (lncRNAs) are a class of RNA transcripts longer than 200 nucleotides that do not encode proteins. Although once dismissed as “genomic junk,” accumulating evidence now establishes lncRNAs as key regulatory players in gene expression through interactions with DNA, proteins, and other RNA molecules, including microRNAs^7,8^. Notably, about 40% of lncRNAs are preferentially expressed in the nervous system, suggesting a close association with neural development, neurological disorders, and NP^9–11^.

lncRNA activity has been extensively investigated in pain-relevant regions, including the spinal cord, dorsal root ganglia (DRG), hippocampus, and prefrontal cortex^11–14^. Deciphering the molecular mechanisms by which lncRNAs contribute to neuropathic pain is therefore no longer solely a basic research endeavor, but represents a promising avenue for the development of targeted and effective therapeutic interventions. For example, lncRNA H19 is upregulated in Schwann cells and spinal cord tissue following nerve injury and exacerbates neuropathic pain by modulating inflammatory pathways, promoting apoptosis, and interacting with specific miRNAs, highlighting its potential as both a biomarker and therapeutic target^12,13^. Similarly, lncRNA Colorectal Neoplasia Differentially Expressed (CRNDE) exacerbates NP in injury-induced models by influencing certain microRNAs, specifically miR-146a-5p^15^ and miR-136^16^.

The functional profile of the long non-coding RNA Growth Arrest–Specific 5 (GAS5) reveals considerable complexity. It is associated with both neuroprotective properties and activating inflammation and apoptosis^17,18^. While some studies have shown that GAS5 silencing and knockdown alleviate inflammation and apoptosis in SCI models and enhance nerve regeneration^19,20^, a recent investigation revealed that elevated expression of GAS5 alleviates NP induced by chronic constriction injury (CCI) in rodent models. This protective mechanism involves regulation of the miR-452-5p/CELF2 signaling pathway and subsequent reduction of inflammatory processes in spinal cord tissue^21^.

MicroRNAs are short RNA transcripts that consist of 18–24 nucleotides, and they present a regulatory role in gene expression at the translational level^22^. The pro-fibrotic microRNA miR-21-5p contributes to fibrotic scar formation and the development of NP following spinal SCI. Conversely, the suppression or knockdown of miR-21-5p has been shown to facilitate motor function restoration^23,24^. Moreover, the upregulation of miR-21 within primary sensory neurons after nerve injuries can contribute to NP in rats^25^.

Recent studies have widely reported that miR-29a dysregulation shows a critical role in processes such as neural development, brain injury, and neurological disorders^26^. miR-29a dysregulation is involved in neural damage and hypersensitivity^27,28^.

Curcumin, the primary bioactive compound derived from Curcuma longa (turmeric), demonstrates potent anti-inflammatory, antioxidant, and neuroprotective activities NP^29,30^. These properties underlie its demonstrated efficacy in mitigating NP^29,30^. Curcumin may exert these effects through various pathways, including the modulation of no-ncoding RNAs^31^. Although curcumin improves motor function after SCI^32^, its effects on central NP following SCI, particularly through the modulation of ncRNAs, remain unexplored. Because neurons of the central nervous system (CNS) have limited intrinsic capacity for regeneration compared to peripheral nerves, therapeutic approaches effective in peripheral neuropathy may not directly translate to CNS injury^30^. Therefore, understanding the underlying molecular and regulatory mechanisms—such as ncRNA networks influenced by curcumin—is critical for the development of new targeted therapies for SCI-associated NP.

The present study aims to investigate the therapeutic potential of curcumin in alleviating neuropathic pain following SCI, with a focus on its modulatory effects on selected key ncRNAs, including lncRNAs (H19, GAS5, CRNDE) and miRNAs (miR-21-5p, miR-29a-3p). Our findings may provide novel insights into RNA-targeted therapeutic strategies for this debilitating condition.

## Materials and methods

### Study design

All methods were carried out in accordance with relevant guidelines and regulations, including the ARRIVE guidelines. The experimental protocol was approved by the Research Ethics Committee of Kerman University of Medical Sciences (Approval Code: IR.KMU.AEC.1401.027). Forty mature male Wistar rats (200–220 g) were obtained from and housed in the Physiology Research Center of Kerman University of Medical Sciences. The experimental animals were housed under controlled environmental conditions, with a 12-hour light/dark cycle, maintained at 22 ± 2 °C and 50–60% relative humidity. All experimental animals were provided with unrestricted access to food and water for the duration of the study. Following an acclimatization period, the animals (200–220 g) were randomly assigned to five experimental groups (*n* = 8 per group): Control group (no surgical intervention or treatment), Sham (lamina removal without SCI), SCI (lamina removal with clip-induced spinal cord compression), Curcumin100 group (SCI + 100 mg/kg curcumin treatment), and Curcumin200 group (SCI + 200 mg/kg curcumin treatment).

To minimize potential stress-related confounders, three rats were housed per cage throughout the study, including the post-operative period. All behavioral assessments were consistently done from 10:00 AM to 3:00 PM to control for potential circadian influences on behavioral outcomes.

### SCI model induction

The SCI model was established following the protocol described by Behroozi et al. Surgical procedures were performed under deep anesthesia induced by a ketamine/xylazine mixture (100 mg/kg and 10 mg/kg, intraperitoneal injection (IP) respectively)^33^. The surgical procedure was performed as follows: The T11-T12 spinal level was identified using the last rib attached to the T13 vertebra as a surgical landmark. the shaved skin and superficial muscles were carefully retracted. The spinal cord was then exposed through a laminectomy at the T11-T12 vertebrae. Severe SCI was induced by applying an aneurysm clip (RS6474, with a force of 35–45 g/cm²; Roboz Surgical Instrument, Gaithersburg, MD, USA) to the exposed cord for 120 s^30^. A similar clip compression model has been previously used in our research network with different closing forces^34–37^. The muscle and skin layers were subsequently sutured in separate layers using 0.3 suture thread.

Postoperatively, manual bladder expression was performed twice daily until reflexive bladder function returned, typically within 7–15 days. Tetracycline spray (OTC, Daru Darman, Iran) was applied to the surgical site to prevent infection, and cages were cleaned daily with 70% ethanol until complete wound healing^30^.

### Preparation and administration of curcumin and experimental timeline

**Fig. 1:**
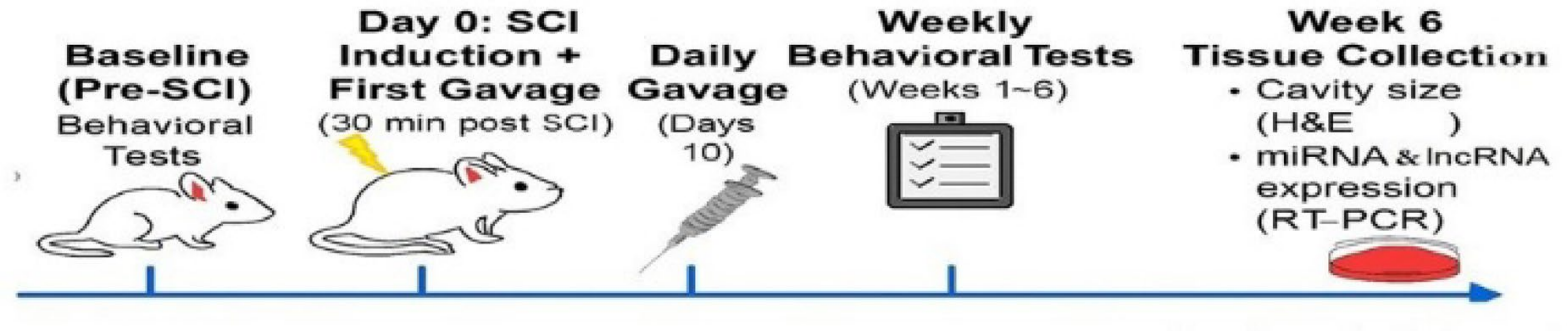
Experimental timeline. Baseline responses to cold (acetone test) and heat (tail-flick test) were assessed 1 h prior to spinal cord injury (SCI). Injury severity was verified 24 h post-SCI using the Basso, Beattie, and Bresnahan (BBB) locomotor scale; only animals with severe injury (BBB score 0–7) were included in the study. Cold allodynia and thermal hyperalgesia were then evaluated weekly for six weeks following SCI. Curcumin (100 or 200 mg/kg) was suspended in 1–1.2 mL of sterile 0.9% saline, homogenized by vortexing (2 min) and sonication (15 min, 40 kHz), and administered once daily by oral gavage for 10 consecutive days, starting 30 min after injury. Animals were sacrificed 1 h after the final behavioral test, and spinal cord tissues were collected for histopathological analysis (H&E staining) and quantitative real-time PCR.

A fresh suspension of curcumin was prepared daily to address its well-documented poor aqueous solubility^38^. Specifically, curcumin powder (Merck Millipore, purity ≥ 80%, Cat. No. 8.20354) was suspended in sterile 0.9% saline to achieve target concentrations of 100 mg/kg and 200 mg/kg. To ensure a homogeneous mixture, the suspension was vortexed vigorously for 2 min followed by sonication in an ultrasonic bath (40 kHz) for 15 min immediately prior to each administration. This method of preparing a stable suspension is consistent with established protocols for administering poorly water-soluble herbal compounds in preclinical studies^39–41^. A schematic timeline illustrating the key stages of the experiment is provided in Fig. 1.

The freshly prepared suspension (1–1.2mL) was administered via oral gavage once daily for 10 consecutive days, beginning 30 min after SCI induction. The administered volume was adjusted daily according to the individual body weight of each animal to maintain the target dose. Sham and SCI control groups received an equivalent volume of vehicle (0.9% saline) on the same schedule.

### Behavioral Tests

All behavioral assessments were conducted two hours prior to SCI induction and then continued weekly for six weeks following the procedure. To ensure objectivity and minimize potential bias, all behavioral tests were performed by two independent researchers who were blinded to the group assignments throughout the testing and data collection process.

Cold allodynia was assessed on the plantar surface of the hind paw, while thermal hyperalgesia was measured on the tail. Both tests are standard assays for NP in rodent models of SCI and reflect sensory processing within spinal segments affected by the T11-T12 injury.

#### Evaluation of motor function

To standardize the injury model, initial lesion severity was verified 24 h post-SCI using the Basso, Beattie, and Bresnahan (BBB) locomotor scale^33^. The scale ranges from 0 (complete paralysis) to 21 (normal locomotor function). Two independent observers, blinded to group assignment, observed each animal’s hindlimb movements for 4 min and assigned scores based on key criteria including joint movement, weight-bearing capacity, paw positioning, limb coordination, and toe clearance. The final score for each animal was calculated as the mean of the two observers’ scores. Only animals with severe injuries (BBB score 0–7) were included in the subsequent pain-related behavioral and molecular analyses, which formed the primary focus of this study^42^. BBB assessment was performed only at 24 h post-injury as an inclusion criterion to ensure consistent injury severity, not as a therapeutic outcome measure.

#### Assessment of acetone-induced cold allodynia

Cold allodynia was evaluated through the acetone drop method. Rats were individually placed in an acrylic enclosure with a wire mesh floor. A single drop of acetone was applied to the plantar surface of each hind paw using a syringe fitted with a blunt plastic needle. Each paw underwent five trials with two-minute inter-trial intervals. Positive nociceptive responses, including paw withdrawal, licking, stamping, leaning toward the affected paw, or jumping, were recorded and quantified^43^. The frequency of both paw withdrawal responses was quantified as a percentage using the following formula: (Number of withdrawals observed/10 total trials) × 100^44^.

#### Assessment of thermal hyperalgesia using the tail-flick test

Thermal pain sensitivity was evaluated using a tail-flick apparatus (Analgesy-Meter LE 7106, Panlab S.L., Spain). The radiant heat intensity was calibrated to a setting of 6. Following a 15-minute acclimation period in a specialized restraint chamber, the heat stimulus was applied to the ventral surface of the distal tail. The tail-flick latency, defined as the time from heat onset to a rapid withdrawal reflex, was automatically recorded. Each animal underwent three trials with a minimum five-minute inter-trial interval to prevent sensitization. The mean latency across trials was analyzed as the nociceptive threshold, with predetermined 15-second cut-off time to avoid tissue injury^30^.

### Tissue harvesting and preparation

At the conclusion of the six-week experimental period, animals from each group (*n* = 8) were deeply anesthetized via administration of ketamine and xylazine at the previously described dose. Tissue was collected in two distinct ways to enable both microscopic (histological) and molecular analyses. For euthanasia, after deep anesthesia, the chest was opened via a midline thoracic incision, and euthanasia was confirmed by cardiac arrest and cessation of respiration. All procedures were performed by trained personnel using sterile equipment.

**For histological analysis:** A subset of animals (*n* = 3 per group) underwent transcardial perfusion. Following a thoracic incision, a perfusion needle was inserted into the left ventricle. The circulatory system was flushed with 0.9% saline, followed by perfusion with 10% neutral-buffered formalin. The spinal cord segment encompassing the injury epicenter (T11–T12) was dissected and post-fixed in the same fixative for 72 h at 4 °C. These samples were then processed following standard histological protocols, including paraffin embedding and sectioning at 4 μm thickness for subsequent staining and cavity size measurement.

**For molecular (qPCR) analysis:** The remaining animals (*n* = 5 per group) were used exclusively for RNA analysis. Immediately after anesthesia, the spinal cord was rapidly exposed. The exact lesion epicenter (T11–T12) was identified, meticulously dissected, and instantly snap-frozen in liquid nitrogen. All samples were stored at − 80 °C until processed for total RNA extraction.

**Rationale for histological sample size:** Given that the primary focus of the study was behavioral analysis (*n* = 8 per group), the histological evaluation of cavity size was designed as a quantitative, correlative confirmatory measure. A sample size of *n* = 3 per group was selected for this histomorphometric endpoint as it aligns with established methodological standards in similar SCI studies and has been demonstrated to be adequate for detecting significant differences in lesion volume when effect sizes are substantial^24,33,45^.

#### Histopathological analysis

H&E staining was performed to verify successful induction of the SCI model and to evaluate the extent of tissue damage by measuring the cavity size at the lesion epicenter. Transverse sections from the rostral, middle, and caudal regions of the injury site were imaged at 4× magnification. The cavity area was quantified using ImageJ software (National Institutes of Health, USA) according to the following formula:

$${\text{Cavity size per section }}\left( \% \right) = {\text{ }}\left[ {{\text{Cavity area }}\left( {\upmu {\mathrm{m}}} \right){\text{ }}/{\text{ Total cross}} - {\text{sectional area }}\left( {\upmu {\mathrm{m}}} \right)} \right]{\text{ }} \times {\text{ 1}}00.$$

The mean cavity size in injury site for each animal was calculated as:

$${\text{Mean cavity size }}\left( \% \right) = {\text{ }}\Sigma {\text{ }}\left( {{\text{Cavity size of rostral}},{\text{ middle}},{\text{ and caudal sections}}} \right){\text{ }}/{\text{ 3}}.$$

#### Quantitative PCR measurement of RNA expression

The expression levels of lncRNA (H19, CRNDE, and GAS5 genes) and miRNAs (miRNA-29a and miR-21-5p genes) were measured using qPCR after extracting total RNA from frozen spinal cord tissue specimens (*n* = 5 per group) using the Total RNA Mini-Preps Kit (ParsTous, Iran). The concentration and purity of the isolated RNA were assessed spectrophotometrically with the NanoDrop 2000c system (Thermo Fisher Scientific, USA). RNA purity was confirmed by A260/A280 ratios between 1.95 and 2.10, and RNA integrity was verified by agarose gel electrophoresis showing distinct 28 S and 18 S rRNA bands. cDNA was synthesized using the PrimeScript 1 st cDNA Synthesis Kit (ParsTous Biotechnology, Iran). For miRNA cDNA synthesis, specific RT primers (Table 1) were included in the reaction mixture.

Quantitative PCR amplification was conducted on the StepOnePlus Real-Time PCR System (Applied Biosystems, USA) employing RealQ Plus 2x Master Mix Green (Amplicon, Denmark). The expression levels of target genes were normalized to RNU6 (for miRNAs) and 18 S rRNA (for lncRNAs). Relative gene expression was calculated through the comparative threshold cycle (2^−ΔΔCt^) method^46^, with the control group as reference:

The following formulas were applied.

For miRNAs:

$$\Delta \Delta {\text{CT }} = {\text{ }}\left[ {\left( {{\mathrm{CT}}\_{\text{target }} - {\text{ CT}}\_{\mathrm{RNU6}}} \right){\text{ }}\_{\text{treatment }} - {\text{ }}\left( {{\mathrm{CT}}\_{\text{target }} - {\text{ CT}}\_{\mathrm{RNU6}}} \right){\text{ }}\_{\mathrm{control}}} \right]$$

For lncRNAs

$$\Delta \Delta {\text{CT }} = {\text{ }}\left[ {\left( {{\mathrm{CT}}\_{\text{target }} - {\text{ CT}}\_{\mathrm{18S}}} \right){\text{ }}\_{\text{treatment }} - {\text{ }}\left( {{\mathrm{CT}}\_{\text{target }} - {\text{ CT}}\_{\mathrm{18S}}} \right){\text{ }}\_{\mathrm{control}}} \right]$$

**Table 1:** Primer sequences used for quantitative PCR analysis.

Gene	Primer Sequence (5’ → 3’)		Ref
miR- 21-5p	Forward: 5′-GCCCGCTAGCTTATCAGACTGATG-3	Reverse: 5′-GTGCAGGGTCCGAGGT-3′	^47^
miR-29a-3p	Forward: 5′-CCGTCCTCCGTAGCACCATCTGAAAT-3′	Reverse: 5′-CTCAACTGGTGTCGTGGAGTCGGC-3′	^46^
RNU6	Forward: 5′-CTCGCTTCGGCAGCACA-3′	Reverse: 5′-AACGCTTCACGAATTTGCGT-3′	^46^
H19	Forward: 5′-GATGGAGAGGACAGAAGGACAGT-3′	Reverse:5′-GAGAGCAGCAGAGATGTGTTAGC-3′	^48^
CRNDE	Forward: 5′-GGGCTTCCCGAGATGTGTTTG-3	Reverse: 5′-CCTTTGCAGTGACTGGGGAAC-3′	^48^
GAS5	Forward: 5′-TCTCACAGGCAGTTCTGTGG-3′,	Reverse: 5′-ATCCATCCAGTCACCTCTGG-3′	^48^
18 S RNA	Forward: 5′-AGTCCCTGCCCTTTGTACACA-3	Reverse: 5′-CGATCCGAGGGCCTCACTA-3′	^46^

Detailed information regarding all materials, reagents, and equipment, including manufacturers and catalog numbers, is available in Supplementary Table S1.

### Statistical analysis

All data were processed using GraphPad Prism version 8.0 (GraphPad Software, San Diego, CA, USA). The normality of data distribution was checked using the Kolmogorov–Smirnov test. For behavioral data with repeated measurements, two-way ANOVA followed by Bonferroni’s post hoc test was applied. Comparisons of cavity size and ncRNA expression among groups were performed using one-way ANOVA with Tukey’s post hoc test. A p-value below 0.05 was considered statistically significant. Results are presented as mean ± standard deviation (SD), and all assessments were carried out under blinded conditions.

## Results

### Curcumin treatment reduces spinal cord injury area

**Fig. 2:**
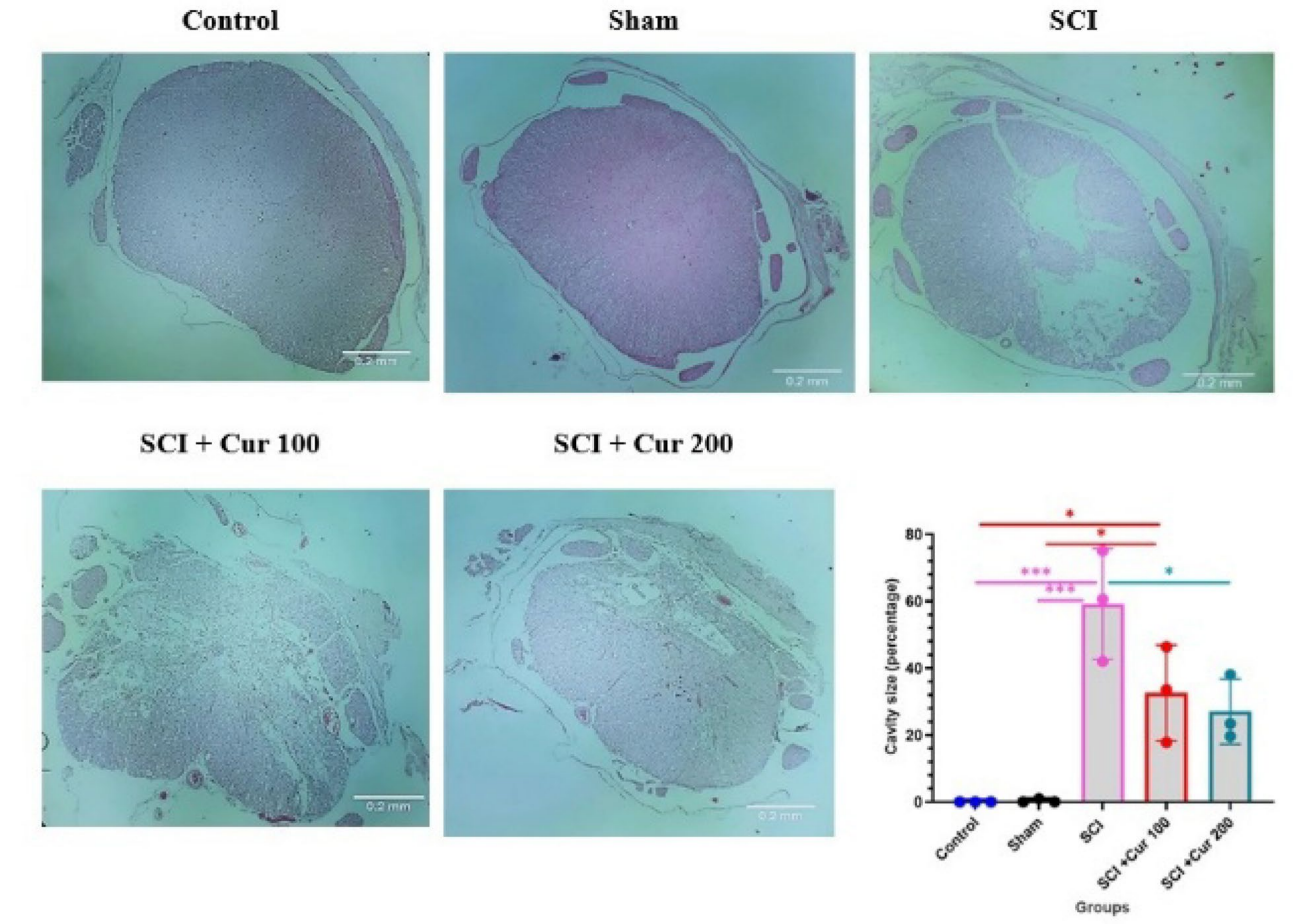
Effect of curcumin on cavity size after SCI. Representative H&E-stained spinal cord sections from each group (**A**). Quantitative analysis of cavity area expressed as percentage of total cross-sectional area Scale bar = 500 μm (**B**). Control (no injury/no treatment), Sham (laminectomy), SCI (Spinal cord compression in the T11-T12 vertebrae using a clip), treated animals with curcumin at doses of 100 mg/kg (SCI+Cur100) and 200 mg/kg (SCI+Cur200). SCI group had the largest cavity size compared to all treatment groups. Curcumin administration, particularly at the higher dose (200 mg/kg), significantly reduced the cavity size, suggesting a neuroprotective effect. Data are presented as mean ± SD (*n* = 3 per group). Data were analyzed by one-way ANOVA with Tukey’s post hoc test. Asterisks indicate significant differences between all groups (*p < 0.05, **p < 0.01, ***p < 0.001). No significant difference was found between Control, Sham, and Cur200 groups.

Statistical analysis of the lesion area revealed significant differences among the experimental groups (F(4,10) = 16.06, *p =* 0.0002; *n* = 3 per group). The largest cavitation was observed in the untreated SCI group (59.26 ± 16.58%; *p =* 0.0004). Curcumin administration at both 100 and 200 mg/kg doses led to a reduction in injury area. However, only the group treated with the higher dose (200 mg/kg; SCI+Cur200) demonstrated a statistically significant reduction relative to the SCI group (27.08 ± 9.75%; *p =* 0.0276) (Fig. 2).

### Behavioral tests

#### Curcumin attenuates cold allodynia

**Fig. 3:**
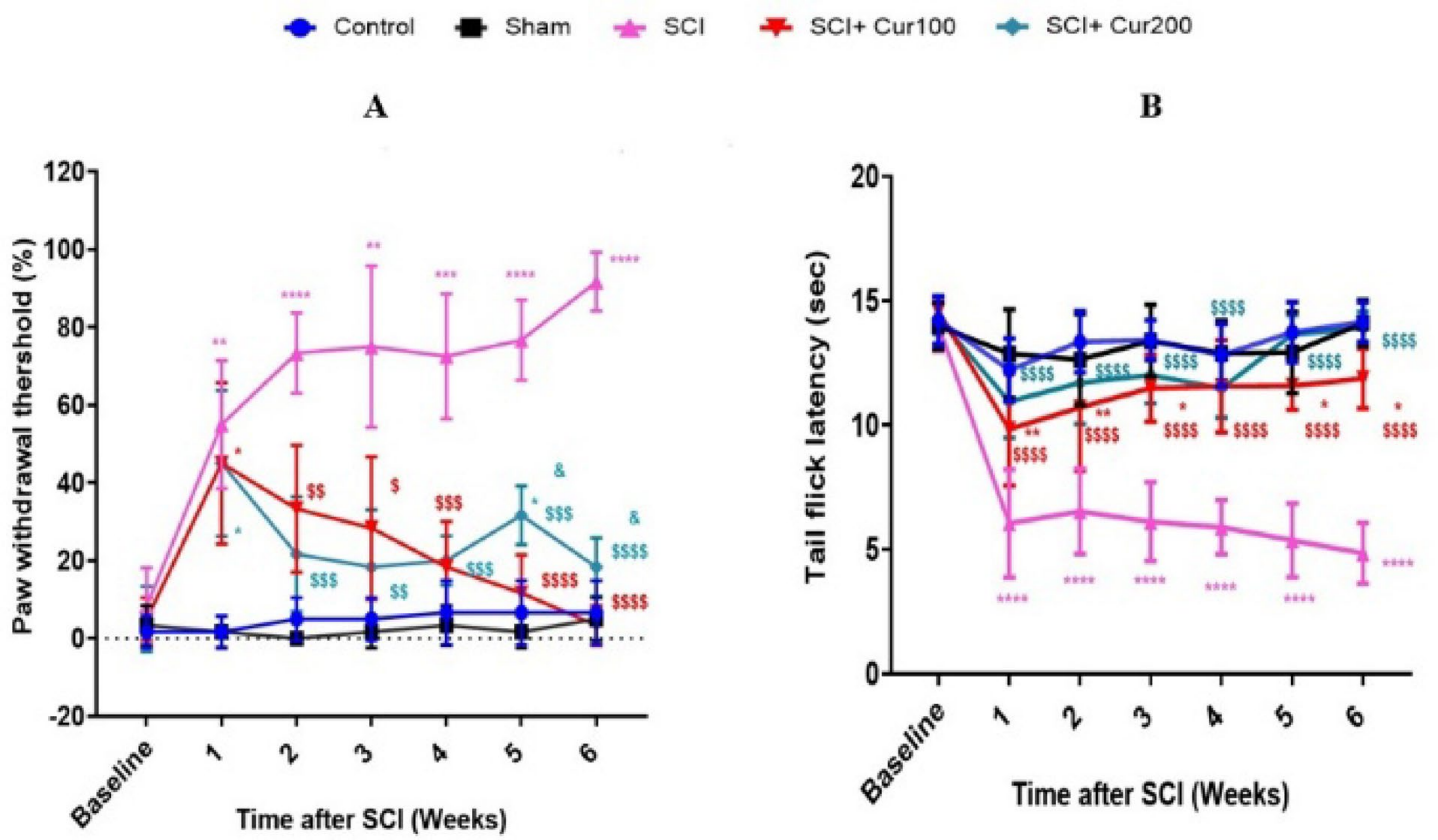
Effect of curcumin on cold allodynia (acetone test) (**A**) and thermal hyperalgesia (tail-flick test) (**B**) following spinal cord injury (SCI). Curcumin treatment at both 100 and 200 mg/kg significantly elevated pain thresholds after SCI. The higher dose (200 mg/kg) was more effective against thermal hyperalgesia, whereas the lower dose (100 mg/kg) was significantly more effective in reducing cold allodynia. Control (no injury/no treatment), Sham (laminectomy only), SCI (clip compression at T11–T12), Cur100 and Cur200 (curcumin at 100 or 200 mg/kg administered orally for 10 consecutive days, starting 30 min post-injury). Data are presented as mean ± SD (*n* = 8 per group). Data were analyzed by two-way ANOVA with repeated measures followed by Bonferroni’s post hoc test. *p < 0.05, **p < 0.01, ***p < 0.001, ****p < 0.0001 vs. Control group; $ *p* < 0.05, $$ *p* < 0.01, $$$ *p* < 0.001, $$$$ *p* < 0.0001 vs. SCI group; & *p* < 0.05 Cur200 vs. Cur100 group. No significant difference was observed between Control and Sham groups at any time point. The absence of a significance marker between any two groups indicates no statistically significant difference.

In the acetone test, animals with SCI showed a higher frequency of paw withdrawal, reflecting increased cold sensitivity, compared to control and sham groups (F(16.83, 105.2) = 10.20, *p* < 0.0001; *n* = 8 per group). Treatment with curcumin at both doses significantly reduced this response from week two onward relative to the SCI group (week 2: *p* = 0.0082 for 100 mg/kg and *p* = 0.0006 for 200 mg/kg; week 6: *p* < 0.0001 for both). During most time points, no significant difference was observed between curcumin-treated and control animals, except for the 200 mg/kg group at week five (*p* = 0.0026). At weeks five and six, the lower dose (100 mg/kg) led to a greater reduction in withdrawal frequency (week 5: 11.67 ± 9.83%; week 6: 3.33 ± 5.17%) than the higher dose (week 5: 31.67 ± 7.53%; week 6: 18.33 ± 7.53%), with the difference between the two doses reaching statistical significance (*p* = 0.030 and *p* = 0.031, respectively) (Fig. 3A).

#### Curcumin attenuates thermal hyperalgesia

Thermal hyperalgesia assessment using the tail-flick test revealed that SCI significantly reduced pain thresholds compared to both control and sham animals. Curcumin treatment demonstrated a dose-dependent therapeutic effect, with both 100 mg/kg and 200 mg/kg doses significantly increasing the hyperalgesia threshold relative to the SCI group (F (24, 245) = 7.180, *p* < 0.0001; *n* = 8 per group). Paw withdrawal latency (pain threshold) was significantly increased in the group treated with curcumin at a dose of 200 mg/kg compared to the SCI group from week one until the end of week six (*p* < 0.0001). However, no significant difference in latency was observed between the 200 mg/kg dose group and the control group throughout all weeks, and at the final week, their levels were nearly identical (Cur200 group: 13.91 ± 0.64; control group: 14.15 ± 0.8).

Although curcumin at a dose of 100 mg/kg, similarly to the 200 mg/kg dose, significantly increased the latency period compared to the SCI group throughout all weeks (*p* < 0.0001), it failed to restore it to the control level in any week. Nevertheless, these values gradually approached control levels over time (*P*-values: week 1: 0.0066; week 2: 0.0017; week 3: 0.045; week 4: NS; week 5: 0.0175; week 6: 0.01).

A significant intergroup differences in the therapeutic effect was detected between the Cur100 and Cur200 groups in weeks five and six, indicating that the 200 mg/kg dose has superior efficacy in alleviating thermal hyperalgesia (Fig. 3B).

### Effect of curcumin on lncRNA expression

#### Curcumin decreased lncRNA H19 expression

**Fig. 4:**
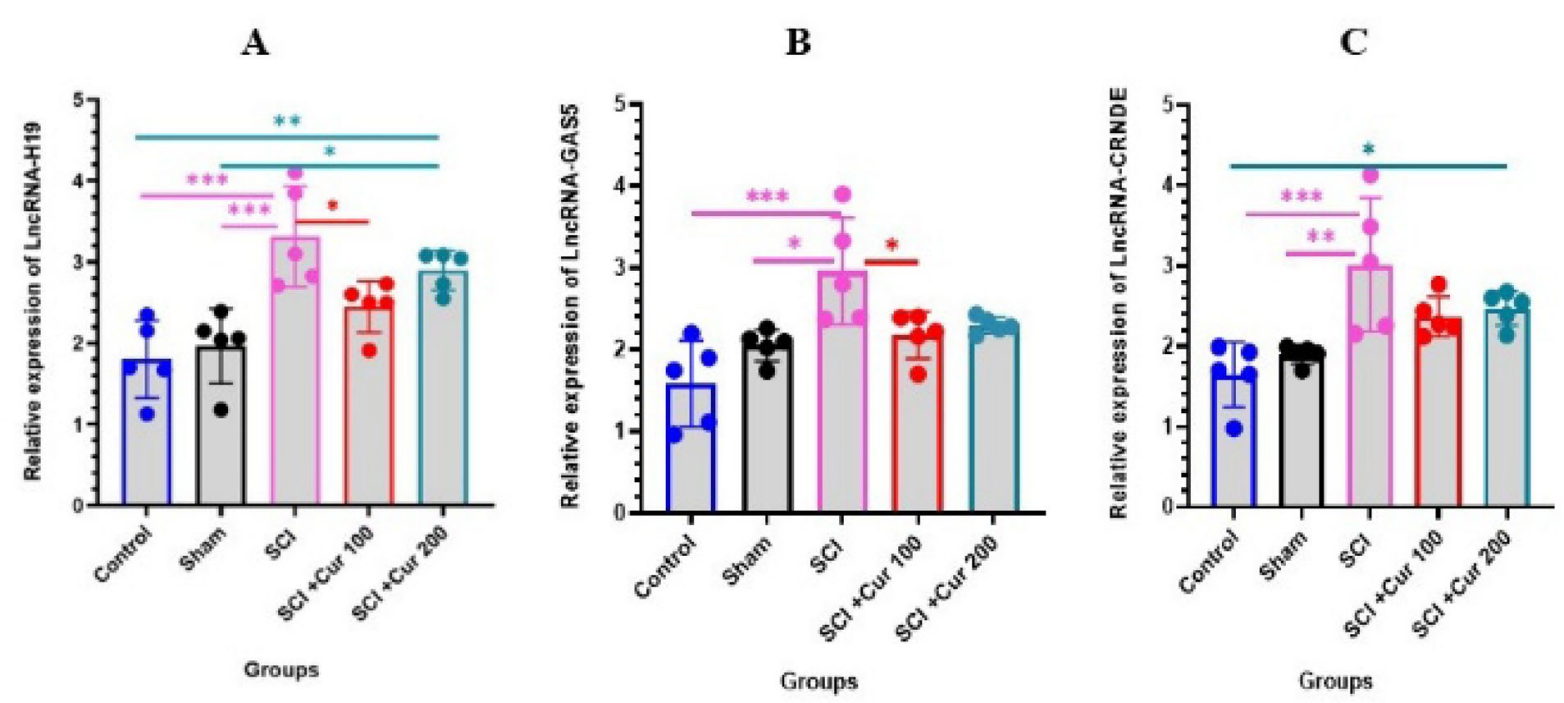
Effect of curcumin on lncRNA expression following SCI. Curcumin (100 mg/kg) significantly reduced SCI-induced upregulation of lncRNA H19 (*p =* 0.04 vs. SCI group) (**A**). Curcumin (100 mg/kg) significantly decreased lncRNA GAS5 expression (*p =* 0.046 vs. SCI group) (**B**). lncRNA CRNDE expression was elevated after SCI but not significantly affected by curcumin treatment (**C**). Data were analyzed by one-way ANOVA with Tukey’s post hoc test and are presented as mean ± SD (*n* = 5 per group). *p < 0.05, **p < 0.01, ***p < 0. 001.The absence of a significance marker between any two groups indicates no statistically significant difference.

Significant differences in lncRNA H19 expression were observed among the experimental groups at six weeks’ post-injury (F (4,20) = 10.18, *p* = 0.0001; *n* = 5 per group). LncRNA H19 expression was significantly increased in the SCI group (3.317 ± 0.623) compared to both normal (1.80 ± 0.475, *p* = 0.0002) and sham (1.96 ± 0.46, *p* = 0.0009) animals. Treatment with curcumin at 100 mg/kg significantly reduced H19 expression (2.448 ± 0.315) relative to the SCI group (*p* = 0.04). However, no statistically significant differences were detected between either curcumin-treated group and the control group (Fig. 4A).

#### Curcumin decreased lncRNA GAS5 expression

Analysis of lncRNA GAS5 expression revealed significant differences among groups (F(4,20) = 7.375, *p* = 0.0008; *n* = 5 per group). At the end of the study, GAS5 expression was significantly elevated in the SCI group (2.96 ± 0.655) compared to control (1.58 ± 0.528, *p* = 0.0003) and sham (2.05 ± 0.193, *p* = 0.0168) animals. While both curcumin-treated groups showed reduced GAS5 levels relative to the SCI group, this reduction reached statistical significance only in the 100 mg/kg treatment group (2.26 ± 0.098, *p* = 0.046) (Fig. 4B).

#### Effect of curcumin on lncRNA CRNDE expression

Analysis of lncRNA CRNDE expression revealed significant differences among the experimental groups at the end of the study (F (4,20) = 7.206, *p* = 0.0009; *n* = 5 per group). lncRNA CRNDE expression was significantly increased in the SCI group (3.015 ± 0.83) compared to normal (1.65 ± 0.40, *p* = 0.0008) and sham (1.897 ± 0.11, *p* = 0.0057) animals. Although both curcumin-treated groups showed reduced CRNDE expression relative to the SCI group, these decreases did not reach statistical significance (Fig. 4C).

### Effect of curcumin on miRs expression

#### Curcumin decreased miR-21-5p expression

**Fig. 5:**
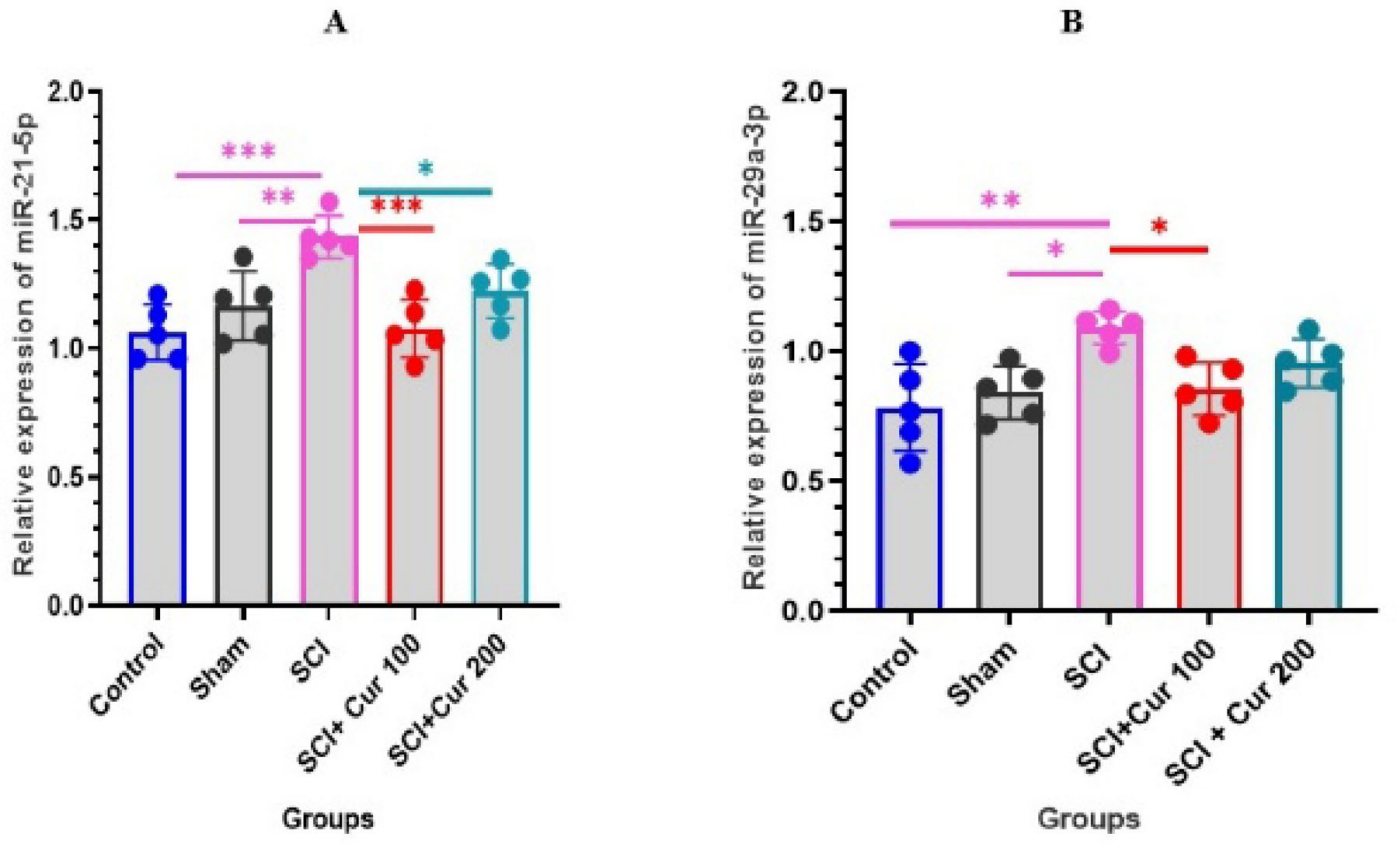
Curcumin attenuates miRNA dysregulation after SCI. Curcumin (100 and 200 mg/kg) significantly reduced SCI-induced elevation of miR-21-5p (*p =* 0.0004 and *p =* 0.045, respectively) (**A**). Curcumin (100 mg/kg) significantly decreased miR-29a-3p expression (*p =* 0.024 vs. SCI group), while the 200 mg/kg dose had no significant effect (**B**). Data were evaluated using one-way ANOVA (Tukey’s post hoc test) and were expressed as mean ± SD (*n* = 5 per group). *p <* 0.05, *p < 0.01, **p < 0.001 vs. SCI group. The absence of a significance marker between any two groups indicates no statistically significant difference. .

Analysis of miR-21-5p expression revealed significant differences among the experimental groups at sixth weeks’ post-injury (F (4,20) = 9.326, *p* = 0.0002; *n* = 5 per group). miR-21-5p expression was significantly increased in the SCI group (1.43 ± 0.083) compared to normal (1.063 ± 0.108, *p* = 0.0003) and sham (1.166 ± 0.134, *p* = 0.0077) animals. Treatment with curcumin at both 100 mg/kg (1.077 ± 0.112) and 200 mg/kg (1.223 ± 0.105) significantly reduced miR-21-5p expression relative to the SCI group (*p* = 0.0004 and *p* = 0.045, respectively). No statistically significant differences were observed between either curcumin-treated group and the control group (Fig. 5A).

#### Curcumin decreased miR-29a-3p expression

Analysis of miR-29a-3p expression revealed significant differences among the five experimental groups at the end of the study (F(4,20) = 5.843, *p* = 0.0028; *n* = 5 per group). miR-29a-3p expression was significantly increased in the SCI group (1.90 ± 0.06) compared to normal (0.784 ± 0.16, *p* = 0.0025) and sham (0.84 ± 0.10, *p* = 0.0159) animals. Treatment with curcumin at 100 mg/kg significantly reduced miR-29a-3p expression (0.855 ± 0.10) relative to the SCI group (*p* = 0.024). However, the 200 mg/kg dose did not produce a significant effect (0.95 ± 0.09, *p* > 0.05). No significant differences were observed between either curcumin-treated group and the control group (Fig. 5B).

## Discussion

Our study revealed several key findings. First, SCI animals exhibited heightened sensitivity to both painful (hyperalgesia) and non-painful (allodynia) stimuli relative to control and sham animals. No significant differences in pain responses were observed between the control and sham groups at any time point, confirming that the laminectomy procedure alone did not induce NP and that the behavioral changes observed were specifically attributable to SCI. These NP hallmarks were associated with a significant upregulation of specific non-coding RNAs (the lncRNAs H19, GAS5 and CRNDE, and miRNAsmiR-29a-3p and miR-21-5p) in the injured spinal cord tissue. Second, post-injury treatment with curcumin at both doses (100 and 200 mg/kg) reduced pain sensitivity and modulated the expression of these ncRNAs. These findings align with previous research. For instance, upregulation of H19 expression has been shown to promote microglial and astrocyte activation, leading to increased production of proinflammatory cytokines IL-1β and IL-6^49^. LncRNA CRNDE has been reported to exacerbate NP in CCI rats through regulation of miR-146a-5p/WNT5A and miR-136/IL6R pathway^15,16^, and its silencing attenuated mechanical allodynia, thermal hyperalgesia, and neuroinflammation^15^.

The functional significance of GAS5 within the nervous system continues to be debated. Our findings align with previous research demonstrating the neuroprotective outcomes associated with its downregulation. Accordingly, silencing or downregulation of GAS5 has been shown to alleviate neural cell apoptosis and inflammation, particularly after SCI^19,20,50^, and its knockout enhances nerve regeneration^20^, potentially through inhibition of the miR-21/PTEN axis^51^.Conversely, other studies have reported decreased GAS5 levels after SCI^52^ or a protective role for its upregulation via the miR-452-5p/CELF2 axis^21^, highlighting its context-dependent function of this lncRNA.

Furthermore, miR-21-5p is associated with fibrotic scar formation after SCI^24,53,54^. Its upregulation in primary sensory neurons following nerve injury has been shown to contribute to NP by promoting pro-inflammatory macrophage polarization in the dorsal root ganglia via exosomal transfer^25,55^. Recent research has consistently demonstrated that miR-29a dysregulation plays crucial roles in neural development, cerebral injury, and neurological disorders. For instance, microRNA-29b-3p has been shown to promote degeneration of terminally differentiated dopaminergic neurons^56^. Regarding miR-29a-3p, its dysregulation plays important roles in brain damage and neurological diseases. Its overexpression may contribute to diabetic peripheral neuropathy^27^ and activation of glutamate receptors—which induces central nervous system (CNS) hypersensitivity— has been elevated miR-29a levels in primary neurons^28^.

The main and novel finding of our study—which emerged unexpectedly during data analysis—is that the therapeutic efficacy of curcumin was significantly parameter-dependent, differing according to the specific anatomical, behavioral, or molecular outcome assessed.

The reduction in lesion size may be attributed to a clearly dose-dependent neuroprotective effect of curcumin. Anatomically, the higher dose (200 mg/kg) provided significantly greater tissue preservation and reduction in lesion size than the lower dose (100 mg/kg). This observation supports a potent, dose-dependent neuroprotective action of curcumin, confirming its ability to limit secondary injury and preserve neural structures at higher concentrations^41^. Moreover, curcumin may exert these effects through various pathways, including the modulation of non-coding RNA^30,31^.

Behavioral analyses revealed that although both doses of curcumin significantly improved overall pain-related outcomes, their efficacy differed according to pain modality. The high dose (200 mg/kg) was superior in reducing thermal hyperalgesia, whereas the low dose (100 mg/kg) was more effective in reducing cold allodynia. We hypothesize that this differential efficacy reflects the distinct neural pathways mediating these pain states. A methodological consideration should be acknowledged here: cold allodynia was evaluated on the plantar surface of the hind paw (acetone test), while thermal hyperalgesia was assessed on the tail (tail-flick test). Both tests are established and widely used in SCI neuropathic pain research. The hind paw is the standard site for assessing cold allodynia due to its high sensitivity to evaporative cooling, and the tail-flick test is a classic, validated method for thermal nociception. Importantly, the T12–T13 vertebral level (L2–L3 spinal cord level) is located within the lumbosacral enlargement (L2–S1), which gives rise to nerves innervating the hindlimbs. The sacral segments, which innervate the tail, are located caudal to this enlargement. An injury at the T12–T13 level directly affects the lumbosacral segments and also interrupts supraspinal input to the sacral segments. Consequently, both hindlimb and tail functions are compromised following the same spinal injury^57^. Furthermore, the observed dose-dependent effects were accompanied by consistent and corresponding molecular changes in spinal cord tissue, supporting that the differential efficacy is biologically meaningful rather than an artifact of the testing site. Nonetheless, we acknowledge this anatomical difference as a consideration when interpreting parameter-specific effects observed in this study. Thermal hyperalgesia is primarily driven by peripheral and spinal sensitization mechanisms that are highly dependent on inflammatory upregulation and the amplification of channels such as TRPV1. Key pro-inflammatory cytokines, including TNF-α and IL-1β, potentiate the activity of TRPV1 and related channels, resulting in amplified noxious heat signaling within the spinal dorsal horn^58,59^. Consequently, the superior effect of high-dose curcumin (200 mg/kg) on thermal hyperalgesia may reflect its potent systemic anti-inflammatory properties, which effectively diminish the peripheral inflammatory cascade^60,61^.

In contrast, cold allodynia involves a more complex central sensitization process arising from the loss of central inhibition and aberrant sprouting of Aβ fibers onto pain-processing spinal dorsal horn neurons, This maladaptive plasticity leads to the misinterpretation of non‑noxious cold signals—mediated by TRPM8 and TRPA1 channels—as painful stimuli^60,62,63^. This central mechanism is significantly influenced by the activation of spinal microglia and subsequent supraspinal processing in regions such as the thalamus^63,64^.

The greater efficacy of the lower curcumin dose (100 mg/kg) against cold allodynia may thus arise from a more targeted and nuanced modulation of the CNS immune response^65^. Rather than inducing broad immunosuppression, the lower dose appears to selectively reduce spinal microglial activation—a key driver of central sensitization^66,67^. This subtle immune modulation disrupts critical proinflammatory signaling cascades (e.g., inhibition of TNF-α, IL-1β, and brain-derived neurotrophic factor (BDNF) release) that facilitate the transmission of maladaptive pain^68^.

Our molecular data provide correlative support for this hypothesis. Low-dose treatment was associated with a downregulation of specific proinflammatory lncRNAs. Particularly noteworthy is the suppression of H19, a lncRNA known to potentiate inflammatory signaling by acting as a molecular decoy for miRNAs that normally inhibit pro-inflammatory pathways^69^. Similarly, the suppression of GAS5, a lncRNA implicated in glial cell activation^17^, and miR-21-5p —a key miRNA that promotes microglial activation and neuroinflammation —^70^ may reflect a precise intervention at both the transcriptional and post-transcriptional levels. By selectively modulating this molecular network, the low-dose curcumin treatment may help normalize the hyper-excitability of dorsal horn neurons and disrupt the aberrant neural circuit rewiring characteristic of cold allodynia, a condition in which non‑noxious cold stimuli are misinterpreted as painful via TRPM8/TRPA1 channels^63^ or through modulation of microglial activation^71^.

Several limitations should be considered when interpreting our findings. First, the sample size for histological quantification (*n* = 3 per group) is relatively small. Although sufficient to detect clear treatment effects, future studies with larger sample sizes would enable more detailed histopathological analysis. Second, curcumin was administered as a saline suspension due to its poor aqueous solubility. Consequently, true bioavailability remains undefined, and the reported doses reflect administered rather than verified tissue concentrations. Future studies should employ bioavailable formulations (e.g., nanoparticles, curcumin‑piperine complexes) with pharmacokinetic validation. Third, our behavioral assessment was limited to thermal and cold pain modalities. The evaluation of mechanical allodynia, a clinically prevalent feature of SCI‑related neuropathic pain, would provide a more comprehensive analgesic profile and is an important direction for future research. Fourth, cold allodynia and thermal hyperalgesia were assessed on different anatomical sites (hind paw vs. tail). Although both are standard assays and the T11–T12 injury innervates both regions, we acknowledge this as a methodological consideration. Future site‑matched testing would further strengthen evidence for parameter‑dependent effects. Fifth, and most importantly, our key finding—the dose‑dependent, parameter‑specific effect of curcumin—was unexpected and emerged post‑hoc. This study was not designed to test this hypothesis or measure TRP channels or inflammatory proteins discussed as possible mechanisms. Therefore, these interpretations remain speculative and require confirmation. This work should be viewed as hypothesis‑generating, providing a foundation for future mechanistic studies.

In conclusion, the differential therapeutic effects of low and high doses of curcumin on behavioral and molecular outcomes suggest that curcumin exerts its effects through distinct, parameter‑dependent mechanisms. Higher doses may be optimal for counteracting inflammation‑driven peripheral sensitization and promoting neuroprotection, whereas lower doses may be particularly effective in modulating specific miRNAs (miR‑21, miR‑29) and lncRNAs (H19, CRNDE) implicated in central sensitization processes such as cold allodynia. These findings underscore the importance of tailoring treatment strategies to the targeted pain modality and molecular pathway. Future studies specifically designed to elucidate the mechanistic basis of this dose specificity—including direct assessment of TRP channels, cytokines, and microglial markers—are warranted to validate and extend the present observations.

## Supplementary Information

Below is the link to the electronic supplementary material.

Supplementary Material 1

## Data Availability

The data supporting this study’s findings can be obtained from the corresponding author following a reasonable request.

## Abbreviations

BDNF: Brain-Derived Neurotrophic Factor
CCI: Chronic Constriction Injury
CNS: Central Nervous System
CRNDE: Colorectal Neoplasia Differentially Expressed
GAS5: Growth Arrest-Specific 5
H&E: Hematoxylin and Eosin
IL-1β: Interleukin-1 Beta
IL-6: Interleukin-6
lncRNA: Long Non-Coding RNA
miRNA: MicroRNA
NP: Neuropathic Pain
qPCR: Quantitative PCR
SCI: Spinal Cord Injury
TBI: Traumatic Brain Injury
TNF-α: Tumor Necrosis Factor-Alpha
TRPA1: Transient Receptor Potential Ankyrin 1
TRPM8: Transient Receptor Potential Melastatin 8
TRPV1: Transient Receptor Potential Vanilloid 1
ncRNA: Non-Coding RNA
