# Supplementary material for: Dose and parameter specific effects of curcumin on neuropathic pain, cavity formation, and non-coding RNA expression in a spinal cord injury model
Source: Sci Rep. 2026 May 13;16:17832. doi: 10.1038/s41598-026-51599-4 (PMC13249888; doi:10.1038/s41598-026-51599-4)
Supplement: Supplementary file 1 — Supplementary Material 1 [file 41598_2026_51599_MOESM1_ESM.docx]

| **Supplementary Table S1. List of materials, reagents, and equipment used in this study** | | | |
| --- | --- | --- | --- |
| **Material/Reagent** | **Manufacturer** | **Catalog No.** | **Description** |
| **Curcumin powder** | Merck Millipore (Sigma-Aldrich), Darmstadt, Germany | C1386 | ≥80% purity, CAS: 458-37-7 |
| **Ketamine 10% solution** | Bremer Pharma GmbH, Warburg, Germany | — | 100 mg/ml injectable solution for anesthesia |
| **Xylazine 2% solution** | Bremer Pharma GmbH, Warburg, Germany | — | 20 mg/ml injectable solution for anesthesia |
| **Aneurysm Clip** | Roboz Surgical Instrument, Gaithersburg, MD, USA | RS6474 | Clip force: 35–45 g/cm²; for SCI induction |
| **Tetracycline spray** | Daru Darman, Tehran, Iran | OTC | Topical antibiotic for post-operative care |
| **Ethanol absolute** | Merck Millipore, Darmstadt, Germany | 100986 | For sterilization and cleaning |
| **Chloroform** | Merck Millipore, Darmstadt, Germany | 102445 | For RNA extraction |
| **Total RNA Extraction Kit** | ParsTous Biotechnology, Mashhad, Iran | A101231 | For isolation of total RNA from tissue |
| **cDNA Synthesis Kit** | ParsTous Biotechnology, Mashhad, Iran | A101161 | For reverse transcription of RNA to cDNA |
| **RealQ Plus 2x Master Mix Green High ROX** | Amplicon, Odense, Denmark | A325402 | For quantitative real-time PCR, 400 reactions |
| **Sonicator** | Hielscher Ultrasonics, Teltow, Germany | UP200H | 50/60 Hz; for homogenization of curcumin suspension |
| **Real-time PCR System** | Applied Biosystems, Foster City, CA, USA | StepOnePlus | For qPCR amplification and detection |
| **Mini Spin Centrifuge** | Eppendorf, Hamburg, Germany | — | For sample preparation |
| **−20°C Freezer** | Bosch, Gerlingen, Germany | — | For short-term sample storage |
| **−80°C Freezer** | Daihan Scientific, Wonju, Korea | — | For long-term sample storage |
| **Thermal Cycler** | Bio-Rad, Hercules, CA, USA | MJ Mini | For cDNA synthesis |
| **NanoDrop 2000c Spectrophotometer** | Thermo Fisher Scientific, Waltham, MA, USA | ND-2000C | For RNA concentration and purity assessment |
